# Predicting driving speed from psychological metrics in a virtual reality car driving simulation

**DOI:** 10.1038/s41598-022-14409-1

**Published:** 2022-06-16

**Authors:** Uijong Ju, John Williamson, Christian Wallraven

**Affiliations:** 1 Department of Information Display, Kyung Hee University, 26-6, Kyungheedae-ro, Dongdaemun-gu, Seoul, 02453 South Korea; 2 Department of Brain and Cognitive Engineering, Korea University, 145, Anam-ro, Seongbuk-gu, Seoul, 02841 South Korea; 3 Department of Artificial Intelligence, Korea University, 145, Anam-ro, Seongbuk-gu, Seoul, 02841 South Korea

**Keywords:** Human behaviour, Personality

## Abstract

Why do some people tend to drive faster than others? Personality characteristics such as the evaluation of risk to oneself or to others, impulsivity, adherence to norms, but also other personal factors such as gender, age, or driving experience all may play a role in determining how fast people drive. Since driving speed is a critical factor underlying accident prevalence, identifying the psychological metrics to predict individual driving speed is an important step that could aid in accident prevention. To investigate this issue, here, we used an immersive virtual reality driving simulation to analyze average driving speed. A total of 124 participants first took a comprehensive set of personality and background questionnaires and a behavioral risk-taking measure. In the virtual reality experiment, participants were required to navigate a difficult driving course in a minimally-restricted, non-urban setting in order to provide baseline results for speed selection. Importantly, we found that sensation seeking and gender significantly predicted the average driving speed, and that sensation seeking and age were able to predict the maximum driving speed.

## Introduction

How fast we drive has direct consequences for traffic safety and accident prevalence: studies have found, for example, that differences in driving speed are related to crash rates in that vehicles that moved faster compared to others had a significantly higher crash rate^1,2^—accordingly, average speed enforcement can be used to reduce the number of (fatal) crash rates^3^. Additionally, data from Farmer et al. showed that the maximum speed is related to fatal accidents in general^4^ and further that a 5 mph increase in the maximum speed limit is associated with an increase in fatal accidents of 8% on freeways and of 4% on other roads^5^.

We also know that driving behaviors related to speed (such as reckless driving, for example) are highly individual across personalities and sociodemographic factors^6,7^, and that accidents related to driving speed such as speeding violations make up a large portion of the total amount of fatal accidents: according to the National Highway Traffic Safety Administration (NHTSA)’s 2020 report, for example, in 2018, speeding accounted for 26% of all traffic deaths in the United States^8^ and the European Automobile Manufacturer’s Association (ACEA) 2019 report indicates speeding to be the cause for 30% of fatal accidents^9^ in Europe. Overall, these studies and reports clearly stress the importance in understanding the factors related to driving speed in order to develop suitable speed management systems based on human factors—for example, intelligent speed assistant systems that implement speed locks for serious speed offenders have been shown to be effective^10^.

In the following, some of the associated personal factors (such as speeding, risky and aggressive driving) known to be *driver-related* are discussed leading to the motivation of the present study.

Several previous studies found that driving speed and resulting effects like speeding were correlated with personality traits and risky and aggressive driving behavior. This includes, for example, sensation seeking^11^, a personality trait that determines how much a person seeks intense, novel experiences and is willing to take risks for them^12^. Studies using questionnaires found that the sensation seeking trait is positively correlated with average driving speed and speeding^13–17^ as well as in exceeding speed limits, resulting in risky and aggressive driving^18–21^.

Another personality trait related to driving speed is impulsivity—a trait referring to those who take immediate action with little or no concern about possible consequences^22^: impulsivity is positively correlated with speeding^23,24^ and risky, aggressive driving^19,20,25^. Furthermore, driving studies found that psychopathy (signaling impaired empathy and disinhibited, egocentric behavior^26^) is associated with speeding^27^, self-reported aggressive driving^28,29^, and involvement in accidents^29^. Finally, Machiavellianism (defined as the trait to pursue one’s own interest by manipulating others^30^) is associated with road-rage^31^ and risky driving attitudes in relation to driving speed^32^, whereas its opposite trait (altruism) negatively correlates with vulnerability to speeding^33^.

Conversely, high anxiety (an emotional state that includes tension, nervousness, and worry^34^) and personal distress (a self-oriented emotional state towards another person’s emotion^35^) have been linked to *decreased* driving speed: specifically, self-reported anxiety traits are associated with aberrant driving behaviors^36,37^, whereas high-anxiety drivers tend to drive with lower speed^38^ and drive more carefully compared to low-anxiety drivers^39^. Additionally, personal distress is known to be inversely related to risk resistance^40^ and psychological aggression^41^ and therefore could also be a potential personality factor leading to decreased driving speed.

As mentioned above, increased driving speed and risky driving are intimately connected in that risky driving tendencies are associated with higher driving speed and speeding violations^39,42,43^. Indeed, previous studies have shown that a *behavioral,* indirect measure of risk-taking—the so-called Balloon Analogue Risk Task^44^—correlates with risky driving behavior^45,46^. In this task, participants are required to pump up balloons with each additional pump yielding reward points. The further the balloons get pumped the higher the risk of them exploding, which for the participant means to lose the reward for this round. In order to avoid this, participants have the option to stop pumping and to bank the reward. The number of pumps across several such trials is used as a risk-taking measure. One may make the analogy between this task and driving, in which higher driving speed with its associated rewards will be balanced against safety^45,46^.

Comprehensive meta reports such as from the World Health Organization (WHO) suggest that driver-related factors like age or gender affect speed^47^; similarly, the US Federal Highway Administration (FHWA)’s report indicates that 44% of all speeding-related crashes involved people under the age of 21 or with driving experience less than 3 years^48^.

Specifically, gender has been examined as a factor affecting increased driving speed, speeding and risky driving: young male drivers, for example, have the tendency to drive more riskily^32,49,50^ while also showing lower concerns of risk compared to female drivers^51,52^. Additionally, male drivers exit the highway faster^53^ and have a tendency for higher speeds in general when compared to female drivers^54^ in the same driving situations.

Similarly, age has been shown to critically affect driving speed, speeding, and risky driving behavior. In this context, young drivers, for example, tend to show higher crash rates^55^. Conversely, speeding as a source for fatal collisions decreases with age^56^ and young adults report that “hurry driving” also decreased with age^57^.

Finally, driving experience needs to be accounted for in examinations of driving speed: previous studies have found that driving experience is related to traffic accidents with young and novice drivers exhibiting higher crash rates^58–60^ but also that the tendency to speed actually increases with driving experience^54^.

Perhaps the most commonly-used method in the aforementioned studies is self-reporting, in which participants are asked about their previous driving behavior, speeding experiences, and attitude towards speeding^13,54,61^. This method, however, is limited by subjective recall errors and a subjectivity bias which could lead to errant data. In fact, several previous decision-making studies found clear discrepancies between self-reported traits and observed actions^62–64^. One method for addressing the issues of self-reports is the use of GPS to record driving speed in real-world environments and has been used to investigate factors of age^65^, or age and personality on risky driving^66^. Although this approach provides detailed data about the actual driving speed, such studies sometimes have a small sample size^61^, and the setup makes it impossible to control for vehicle type, traffic flow, or weather conditions during the recording period, potentially limiting the generalizability of the results.

Since none of the previous methods allow controlling traffic environmental factors that may influence driving speed, recent studies have used driving simulations as their main investigation paradigm. For example, Schmid et al.^67^ showed that priming of masculinity significantly increased driving speed in a driving simulation. Two other studies examined the influence of impulsivity on risky driving behavior and found that high-risk drivers showed higher impulsivity traits and faster driving speed^25,46^. Additionally, recent studies found that sensation seeking is significantly correlated with average driving speed^13–17^ and gender^68^, driving experience^69^, and age^17^ influencing driving speed.

However, to the best of our knowledge, driving simulator studies have rarely investigated driving speed in minimally-restricted or -surveilled environments (such as rural areas except rural highways^14,68^) and *directly* tried to associate the driving speed with personal factors. Based on NHSTA’s 2020 report, in 2018, whereas only 19% of the population lived in areas, 45% of fatal accidents occurred in rural areas with 26% of fatalities in rural areas related to speeding^70^. Furthermore, none of the prior studies investigated associations between driving speed and an actual decision made in a high-risk event (such as an accident)—this is important as we do know that risky driving behavior and faster driving speed are associated^25,46^ and hence investigating decision-making in a high-risk event is a potential method to determine risky driving behavior.

Given the importance of absolute and relative speed levels and the fact that driving behavior is different from individual to individual, here we investigate whether it is possible to predict a person’s driving speed from various personal background factors (including a behavioral risk-taking measure, the so-called Behavioral Analogue Risk Task (BART)^44^) in a driving simulation set in a non-urban environment. In the current study, we present the first data from experiments on this question, in which we examined speed predictability in the absence of other traffic on an otherwise empty, but challenging driving course similar to the driving environment on rural roads.

For this, we used an immersive driving simulation in which participants had to navigate a difficult course and measured the average and maximum driving speed of the driver as well as their decision in a high-risk, accident situation—in this situation, drivers were just rounding a corner at high speed towards a fork, when they were suddenly forced to choose between collision with trees blocking one path of the fork, or steering their car down a cliff that followed the other path. In a separate, pre-experiment session, we also measured six personality traits and one indirect risk-taking measure as well as other personal factors, such as gender, age, and driving experience. In our experiment, we specifically focused on two adult groups between 17 and 24 years (a group that had more speed violations^56,71,72^) contrasted with an older group of 24–31 years of age. Statistical tests and multiple regression models were then used to identify those personal factors that significantly predicted the measured driving speeds. To summarize, the aim of the present study was to employ virtual reality (VR) to investigate whether it is possible to predict the average and maximum driving speed in a rural road environment from different psychological metrics.

## Materials and methods

### Dataset

Data were derived from our previous VR decision-making study^73^ and additional data were acquired from an ongoing pilot experiment investigating audio driving instructions^74^.

### Equipment

**Figure 1:**
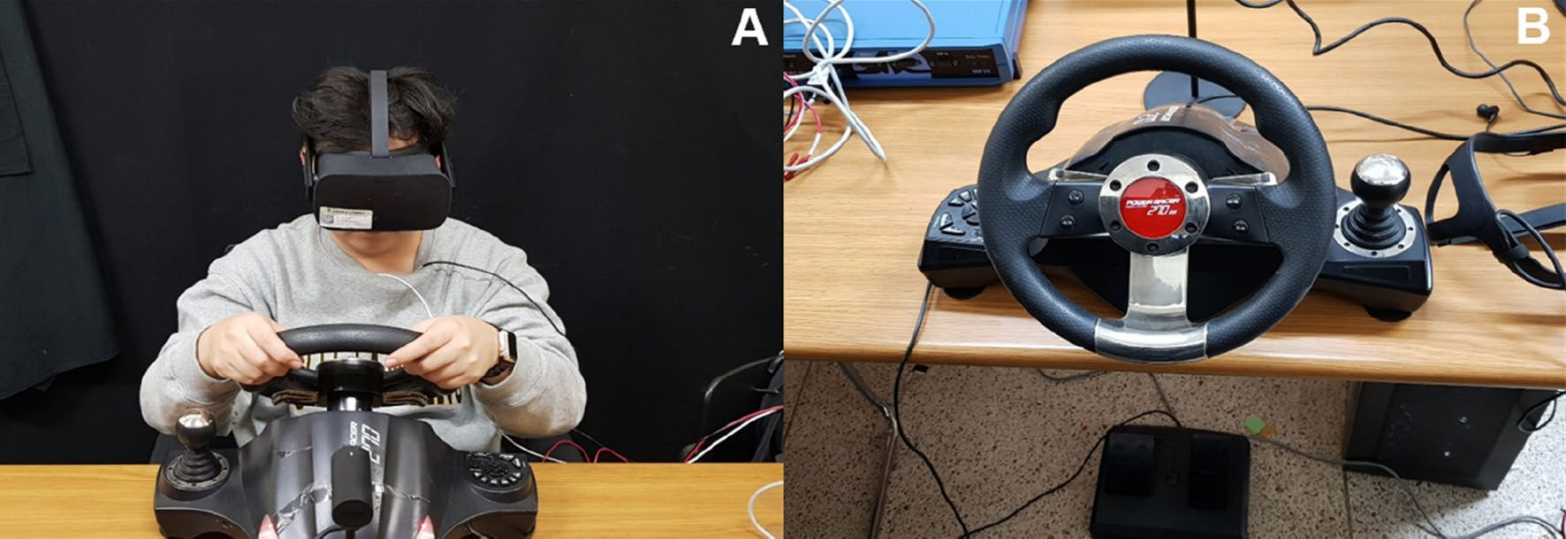
Equipment and setup of the experiment (**A**) frontal view of experimental setup (**B**) closed view which show wheel-pedal interface.

VR visualization was presented through a head mounted display (HMD, Oculus Rift CK1; Irvine, USA; resolution = 1080 × 1200 px for each eye at 90 frames per second), and a wheel-pedal interface (Joystick, Power Racer 270 DX; Seoul, KR) was employed to provide realistic car controls with two degrees of freedom from the wheel and foot controls (see Fig. 1).

### Game design

**Figure 2:**
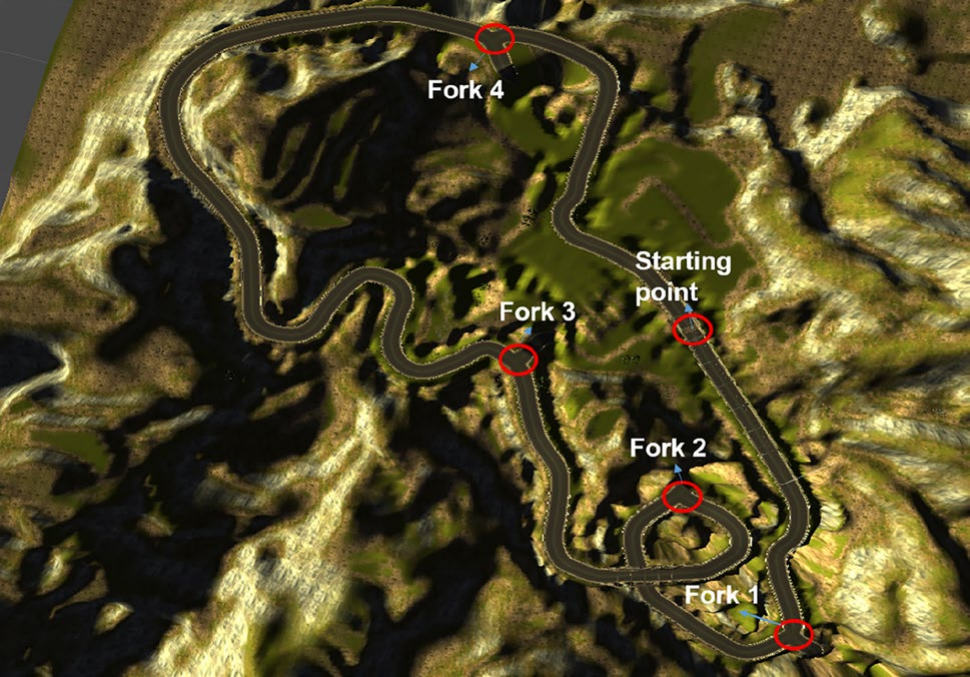
Birds-eye view of driving course with staring point and four crossroads indicated with red circles and blue arrows.

Using a virtual reality development tool, Unity3D 2017.2.0.f3 (Unity Technologies, San Francisco, USA), we created a car-driving simulation from both freely available assets and commercially purchased assets. The basic layout of the driving course was taken from (https://www.assetstore.unity3d.com/en/#!/content/10), to which we added multiple forks and obstacles using a purchased asset (easyroad3d pro: https://assetstore.unity.com/packages/tools/terrain/easyroads3d-pro-v3-469, see Fig. 2). Furthermore, we added the driving capabilities based on a purchased car asset (realistic car HD: https://assetstore.unity.com/packages/3d/vehicles/land/realistic-car-hd-01-135301) and used human avatars and animations from another, purchased asset (population system pro: https://assetstore.unity.com/packages/3d/characters/population-system-pro-59820). In order to ease the task to a moderate level of difficulty, we removed the gear shift and utilized the brake pedal also as a backward accelerator (e.g., when backing up). The maximum speed of the vehicle was limited to 140 km/h, which encompasses most world-wide motorway speed limits^75^ except for the German autobahn and the motorways in the UAE. Car engine and navigation sounds were presented through the VR headset’s earphones to further increase immersion in the driving environment.

**Figure 3:**
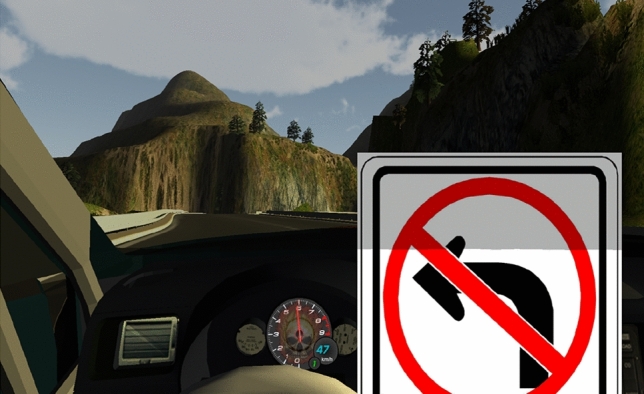
Screenshot of the warning signal given to participants just before one of the forks.

The present study consisted of three training sessions and one test session. Before entering the training session, participants were informed that the aim of the training was to learn how to drive in VR as well as to adjust to the challenging course layout. The course contained multiple forks where at each location, one (randomly chosen) direction led to a cliff—if the car entered in this direction this would lead the participant to fail this session. Additionally, participants were informed that in order to move to the final test session they would need to finish at least one full course without failing. In order to prevent falling down the cliff, visual and auditory warnings were given before each fork that informed the participant of the cliff’s direction. The visual signal was a standard cliff-danger sign and the auditory warning signal was a human voice announcing the direction of the cliff (see Fig. 3). The auditory announcement was made in the Korean language using the Mac OSX built-in “Yuna” voice, e.g.: “Cliff to the left.” At typical driving speeds, the course would take around 2 min 20 s to finish.

Before entering the test session, participants were reminded that the lap time in the next run (the test run) would be recorded and that if they failed to reach the goal, they would have to try again. During the test session, an unexpected, sudden event occurred (pedestrians crossing the road or falling trees appeared) on the side of the fork *without* the cliff. This was done to investigate decision-making in a high-risk event, and the experiment automatically finished after the participants chose their direction at the fork. As shown in^73^, a risky decision would consist of taking the turn leading to the cliff. Its connection to the driver’s personality characteristics is analyzed further in^73^ and is not the focus of the present study. Instead, here we focus on analyzing both the average and maximum driving speed of the car prior to the event and their connection to the decision (in addition to the personal background factors—see below).

Participants were also informed that if they felt dizziness or motion sickness during the experiment, they should immediately take off the headset and stop the experiment.

### Sample size and participants

For the present study, we calculated the required sample size using power analysis (G* power^76^) based on a linear multiple regression model. In order to set the required *η*^2^ value, we referenced prior work that found significant correlations between personality factors (sensation seeking, anger) and speeding behavior^13^. Their study 1 found *η*^2^ = 0.179 for six factors and *η*^2^ = 0.116 for ten factors, respectively, using a linear multiple regression with a sample size of n = 143. We therefore opted for a medium effect size of *η*^2^ = 0.15 with a standard power of 1 − ß = 80% and α = 0.05—the power level follows recommendations for driving simulator studies^77^. As we sought to correlate seven different personality scales (psychopathy, Machiavellianism, sensation seeking, impulsivity, anxiety, personal distress, and results of the BART paradigm—see below) and three personal factors (age, gender, annual driving distance) and one driving behavior factor (risky decision-making in the event), the number of predictors was set to 11, resulting in a required sample size of n ~ 123 for the desired statistical power.

We therefore invited a total of 124 participants for our experiment (93 male, 31 females, between 19 and 37 years of age with a mean age of 23.3 years (SD = 2.74)). All of the participants were Korea University students and recruited from an online advertisement on the Korea University community site. All participants possessed a proper driving license and had normal or corrected normal vision. None of the participants reported any neurological or psychological disease.

### Personality questionnaires

Based on the review outlined in the introduction, we chose the following personality scales potentially associated with speeding and/or risky driving: sensation seeking^11^, the Barratt impulsiveness scale^78^, Levenson's self-report psychopathy scale^79^, Machiavellianism^80^, personal distress (a sub-scale of the interpersonal reactivity index^81^), and anxiety^82^.

In our experiment, we used validated, Korean versions of sensation seeking^83^, impulsivity^84^, psychopathy^85^, Machiavellianism^86^, anxiety^87^, and the inter-personal reactivity index^88^.

### Other personal factors

Since the literature review showed that age, gender, and driving expertise may influence driving behavior, we divided participants into two groups based on age (below and above 25), gender (male and female), and driving frequency (drove in the past year versus did not).

### Risk-taking measurements

The first behavioral risk-taking measure was taken as the decision during the final event as explained above. Second, based on previous findings by^46^, we measured the risk-taking tendencies of the participants using the balloon analogue risk task (BART^44^). Parameters set for this paradigm were as follows: there were a total of 30 trials in each of which the balloon was randomly set to explode between 1 and 128 pumps drawn from a uniform distribution. Participants would receive 2 points for each pump, which they were able to keep only if they stopped pumping before the balloon exploded. During the experiment, the current potential reward for each trial and the total reward gained so far were presented on screen. The total adjusted pump score^44^ was taken as the final BART measure.

### Procedure

A detailed account of the experimental procedure can be found in^73^ and explained here again: 2–4 days before the main VR driving experiment, participants conducted a pre-test, filling in the personality questionnaires, and performed the BART task with the same VR equipment that was used in the main task. Prior to the main experiment, the experimenter explained the task to participants, where they were to use the wheel-pedal interface to drive a car in VR, with the ultimate aim of the study being the investigation of their driving behavior in the VR context. Additionally, participants were informed that their lap times for the test session would be recorded. Furthermore, they were told that during the experiment, it may be possible to fail to reach the finish line if they entered a fork in the wrong way (i.e., choosing the turn leading to the cliff) and that in order to advance to the test session, they would need to finish at least one training session without failing. There were a total of three training sessions and after each training session, the experimenter checked the condition of the participants and asked whether they would be able to start the next training session. After the final session, the experimenter told participants that the next session would be the final, test session and that their lap time would be recorded. After the VR session was over, participants answered additional questionnaires related to the event.

Table 1 provides general information about the participants and their performance on the tasks. Here, we focus on reporting of the final test session results with results from the training sessions analyzed in the “Supplemental results”.

**Table 1:** Descriptive statistics of participants, personality scales are rescaled to 1–100% for easier comparison.

	Descriptive statistics	Skewness
Gender male/female	93/31	0.592
Age (min–max/std)	23.30 (19–37/2.74)	1.169
Age 25 over/under	35/89	0.979
Driving frequently—yes/no	62/62	0
Risky decision-making in the event—yes/no	50/74	− 0.399
Annual driving distance: km/year (min–max/std)	1055.66 (10–15,000/2794.294)	3.260
BART (min–max/std)	27.08 (4–64/11.76)	0.592
Average driving speed (test)—km/h (min–max/std)	64.71 (29.34–82.60/10.09)	− 0.500
Maximum speed (test)—km/h (min–max/std)	117.66 (60–152/18.01)	− 0.850
Psychopathy (min–max/std)	34.58 (13.46–64.42/9.10)	0.312
Personal distress (min–max/std)	41.82 (0–86/17.01)	0.124
Sensation seeking (min–max/std)	52.72 (20–80/12.60)	− 0.335
Machiavellianism (min–max/std)	42.69 (18–83/12.69)	0.382
Anxiety (min–max/std)	40.10 (3–74/10.61)	0.451
Impulsivity (min–max/std)	34.08 (9–62/10.32)	0.331

### Ethical statements

Informed consent for both legal guardian/s for publication of identifying information/images in an online open-access platform and study participation was obtained from all participants before the experiment.

### Ethics approval

The study was approved by Korea University’s local ethics committee (KU-IRB-16-127-A-1). All methods were carried out in accordance with relevant guideline and regulations.

## Results

### Descriptive analysis

Summary statistics of the full sample of 124 participants are shown in Table 1.

To assess the inter-correlations between personal factors, we evaluated Pearson correlations between personality traits, age, annual driving distance, BART scores and risky decision-making in the event situation (see Table 2).

**Table 2:** Correlations between driving experience, personality traits, age, gender, BART score, and risky decision-making.

	D_1_	X_1_	X_2_	X_3_	X_4_	X_5_	X_6_	P_1_	P_2_	R_1_
D_1_ driving distance										
X_1_ psychopathy	0.06									
X_2_ personal distress	− 0.02	0.11								
X_3_ sensation seeking	0.02	0.12	− 0.10							
X_4_ machiavellianism	− 0.02	0.74**	0.05	0.11						
X_5_ anxiety	− 0.14	0.36**	0.36**	− 0.03	0.41**					
X_6_ impulsivity	− 0.10	0.46**	0.28**	0.27**	0.37**	0.29**				
P_1_ age	0.09	− 0.09	− 0.12	0.05	− 0.12	− 0.02	− 0.02			
P_2_ gender	− 0.09	− 0.08	0.47**	− 0.17	− 0.18*	0.21*	− 0.06	− 0.08		
R_1_ BART	0.11	0.02	− 0.06	0.17	< 0.01	− 0.06	< 0.01	− 0.13	− 0.13	
R_2_ decision-making	0.14	− 0.26**	− 0.05	0.21**	− 0.25**	− 0.13	− 0.05	0.02	− 0.02	0.19*

*p < 0.05.

**p < 0.01.

Results showed significant inter-relations for psychopathy (with Machiavellianism (r = 0.74, *p* < 0.001), anxiety (r = 0.36, *p* < 0.001), impulsivity (r = 0.46, *p* < 0.001), risky decision-making (r = − 0.26, *p* = 0.004)), personal distress (with anxiety (r = 0.36, *p* < 0.001), impulsivity (r = 0.28, *p* = 0.002), gender (r = 0.47, *p* < 0.001)), sensation seeking (with impulsivity (r = 0.27, *p* = 0.003), risky decision-making (r = 0.21, p = 0.018)), Machiavellianism (with anxiety (r = 0.41, *p* < 0.001), impulsivity (r = 0.37, *p* < 0.001), risky decision-making (r = − 0.25, *p* = 0.006)), anxiety (with impulsivity (r = 0.29, *p* = 0.001), gender (r = 0.21, *p* = 0.019)) and the BART score (with risky decision-making (r = 0.19, *p* = 0.035)). Other personal factors like annual driving distance or age were not significantly correlated with personality traits or personal factors (see Table 2 for full results); interestingly at first glance, the overall BART score was not significantly correlated with sensation seeking—this finding, however, is well-known and the correlation we obtained was of similar strength to that reported in^89^.

### Effects of gender, age, and driving experience

Next, we investigated group differences in speed selection depending on the factors of gender, age, and driving experience (Table 3).

**Table 3:** Descriptive statistics for male and female participants, age over 25 and under 25, driving frequently or not.

	Male	Female	Age under 25	Age over 25	Infrequent drivers	Frequent driver
Age (std)	23.42 (2.60)	22.94 (3.16)	**22.00 (1.56)**	**26.60 (2.28)**	22.76 (2.92)	23.84 (2.45)
Annual driving distance—km	1203.08 (2881.39)	625.97 (2411.18)	1165.51 (3068.68)	787.46 (1828.77)	**0**	**2117.60 (3637.16)**
BART (std)	27.95 (10.82)	24.47 (14.10)	28.37 (12.04)	23.81 (10.50)	27.46 (12.55)	26.70 (11.01)
Risky decision-making in the event—yes/no	38/55	12/19	34/55	16/19	20/42	30/32
Av. driving speed (test)—km/h (std)	**67.11 (9.32)**	**57.51 (8.95)**	64.89 (9.99)	64.25 (10.49)	**62.19 (10.48)**	**67.23 (9.09)**
Maximum speed (test)—km/h (std)	119.81 (16.06)	111.23 (21.94)	**120.13 (16.83)**	**111.37 (19.58)**	115.16 (19.48)	120.16 (16.18)

Significant differences (p < 0.05/3) between the two groups are shown in bold.

First, to investigate the effect of gender on our main question, we evaluated driving speed differences between male and female participants of the sample n = 124 participants with independent sample t-tests (Bonferroni corrected at α = 0.05/3). The results show that males drove significantly faster in the test session on average (independent sample t-test: *t*(122) = 5.017, *p* < 0.001, *Hedge’s g* = 1.04) and also had tended to have a higher maximum driving speed (independent sample t-test: *t*(122) = 2.339, *p* = 0.021, *Hedge’s g* = 0.48). However, other personal factors like age, annual driving distance, BART scores, or risky decision-making were not significantly different between male and female participants.

Second, we investigated the effect of age on average and maximum driving speed between age over 25 and under 25. While there were no significant differences in average driving speed (independent sample t-test: *t*(122) = 0.318, *p* = 0.751, *Hedge’s g* = 0.06), the younger group had a significantly higher maximum driving speed (independent sample t-test: *t*(122) = 2.490, *p* = 0.014, *Hedge’s g* = 0.50).

Finally, we investigated the effect of driving experience and found that there were significant differences in average driving speed (independent sample t-test: *t*(122) = 2.866, *p* = 0.005, *Hedge’s g* = 0.51), but none in maximum driving speed (independent sample t-test: *t*(122) = 1.555, *p* = 0.123, *Hedge’s g* = 0.28).

Overall, our results showed that gender, age, and driving experience had effects associated with average or maximum driving speed (see “Supplemental results” for more detail).

### Predicting the average driving speed from personal factors

Next, we conducted a multiple linear regression analysis to determine how a combination of personal factors would be able to significantly predict the *average* driving speed. The overall regression model with 11 predictors was significant with r^2^ = 0.29, which corresponds to at least a “moderate effect” (r^2^ > 0.25 according to standards in the psychology literature^76^). Looking at individual contributions within this model, we found that the two factors of sensation seeking (β = 0.24, *p* = 0.009) and gender (β = − 0.40, *p* < 0.001) significantly predicted average driving speed during the test (see Table 4)—males drove faster than females and individuals with a higher sensation-seeking score also drove faster.

**Table 4:** Regression analysis for average driving speed during the test session. Upper and lower 95% confidence intervals are shown in the third and fourth columns.

	Driving speed—test				
	Unstandardized coefficient β	Low CI	High CI	Standardized coefficient β	Statistical significance p
Psychopathy	0.144	− 0.126	0.415	0.135	0.293
Personal distress	0.170	− 0.252	0.593	0.080	0.426
Sensation seeking	0.472	0.123	0.821	0.236	0.009**
Machiavellianism	− 0.033	− 0.368	0.301	− 0.025	0.843
Anxiety	0.028	− 0.155	0.211	0.029	0.765
Impulsivity	− 0.107	− 0.322	0.108	− 0.099	0.326
Annual distance (km/h)	− 0.00002	− 0.001	0.001	− 0.006	0.947
Age	− 0.080	− 0.686	0.526	− 0.022	0.794
Gender	− 9.362	− 13.837	− 4.887	− 0.403	< 0.001**
Risky decision-making	3.118	− 0.469	6.705	0.152	0.088
BART	0.079	− 0.063	0.222	0.092	0.272

*p < 0.05.

**p < 0.01.

### Predicting the maximum driving speed from personal factors

Similar to the average driving speed, we used all personal factors in a multiple linear regression analysis to predict the *maximum* driving speed during the test sessions. First, the regression model revealed a significant contribution of 11 predictors with an overall, moderate effect size of r^2^ = 0.24. Second, we found that sensation seeking (β = 0.36, *p* < 0.001) and age (β = − 0.19, *p* = 0.032) significantly predicted driving speed during the test session (see Table 5 for detailed results) –maximum speed was higher for individuals with a higher sensation seeking score and for younger drivers compared to older ones.

**Table 5:** Regression analysis for maximum driving speed during the test session. Upper and lower 95% confidence intervals are shown in the third and fourth columns.

	Maximum driving speed—test				
	Unstandardized coefficient β	Low CI	High CI	Standardized coefficient β	Statistical significance p
Psychopathy	0.008	− 0.492	0.508	0.004	0.975
Personal distress	− 0.002	− 0.783	0.778	− 0.001	0.995
Sensation seeking	1.275	0.631	1.919	0.357	< 0.001**
Machiavellianism	0.093	− 0.525	0.712	0.039	0.765
Anxiety	0.092	− 0.246	0.430	0.054	0.592
Impulsivity	− 0.268	− 0.665	0.129	− 0.138	0.184
Annual distance (km/h)	0.000	− 0.001	0.001	0.037	0.675
Age	− 1.229	− 2.348	− 0.110	− 0.187	0.032*
gender	− 6.611	− 14.870	1.649	− 0.160	0.116
Risky decision-making	4.569	− 2.051	11.189	0.125	0.174
BART	0.078	− 0.185	0.340	0.051	0.560

*p < 0.05.

**p < 0.01.

## Discussion

The present study tried to establish correlations between different psychological metrics (personality traits, personal factors, and risk-taking measures) and the resultant driving speed in a VR driving simulation. Based on regression analysis, we found that sensation seeking was a crucial, strong predictor for both average and maximum driving speed. In addition, gender was able to predict the average speed, and age was able to predict the maximum driving speed.

Overall, our findings support previous studies that determined sensation seeking as a factor for predicting speed both post-hoc^13,54,56^ and in driving simulations^14–17^ in addition to aggressive and risky driving^18–21^. Several studies have found that higher sensation seekers tended to underestimate danger and took more risk while driving^20,29^, which potentially led to increased driving speed. Importantly, the present study extends these results to a minimally-restricted, rural driving situation and found that average driving speed and maximum driving speed can be predicted from psychological metrics.

In addition, we found that gender was another crucial factor to predict *average* driving speed with male drivers being ~ 17% faster than female drivers. Similar results can be found in the literature for male drivers having the tendency to drive faster^53,54^ and to take more risks^18–21^. Interestingly, gender predicted average driving speed but not maximum driving speed. One potential explanation for this result could be the smaller effect size for maximum speed (see Sect. 3.2)—nonetheless, the regression coefficient of the model did point in the same direction (see Tables 4 and 5).

Additionally, we found that age significantly predicted maximum driving speed with older participants having a slower maximum speed value—comparing the age ranges of 19–24 years with those 25 years and older, maximum speed dropped around 7%. Previous studies have found that younger drivers were more likely to engage speeding related fatal collisions^56^ or “hurry driving”^57^ and from the investigation of drivers from all ages, around 40% of drivers who repeatedly speeded more than 30 km/h were between 17 and 24 years old^71,72^. Additionally, adults between the years of 21 and 24 exhibited a higher intention to exceed the speed limit, which decreased as age increased further^56^. These results imply that maximum driving speed would first increase with age in young adults and decrease again after age 25, which matches to our data.

Importantly, other factors—including the personality traits of psychopathy, anxiety, impulsivity, Machiavellianism, driving experience, as well as risky decision-making during driving and BART—did not significantly predict driving speed. This implies that the effect of these factors on the driving speed are weaker than the influence of the other personal factors mentioned above.

The present study measured driving speed in a simulation without traffic flow or an explicit, instructed speed limit (but with a vehicle speed limit)—something that almost all real-world driving contexts provide. This omission was on purpose to simulate a minimally-restricted, rural driving environment. Future studies will need to be done to extend the driving contexts to include other traffic as well as instructed speed limits in different types of scenarios (urban, rural, or highway).

The results we obtained also were gathered from an age-limited sample of Korean drivers that contains a male bias. In this context, it is important to note that annual accident reports show that in Korea, accident rates of male drivers were three times higher compared to female drivers^4^ and that male drivers under 25 in general tend to have an increased risk for accidents^5^—hence, our sample contains a composition similar to this overall bias, focusing on more “accident-prone” sub-populations. In addition, our data fits with the general trajectory of speed preferences found in other studies in (younger) adults^23,56,57,71,72^, however, additional data with a wider age-range is needed to more fully capture the effects of age on speed preferences (see “Supplemental results” for additional analyses of the results split by gender and age). Similarly, such a wider age-range would also allow us to more closely investigate the effects of driving experience: here, we found differences in average driving speed depending on driving frequency, whereas this factor did not come out in the regression. This result is somewhat in contrast to the literature where speeding tendency increased with driving experience^54^, but may be explained by the large variability in experience contained in our sample. As Korean drivers tend to buy cars later in life compared to other countries^90^, a wider age-range may be necessary to clearly capture effects of driving expertise/experience.

Finally, previous studies have shown that there are cultural differences for speed choices, speeding, or the attitude towards speed limits^91,92^, and it is not inconceivable that the pattern of how personal factors influence driving speed may also be different across cultures. Although it will be interesting to test our experiment in samples with different cultural background, we want to point out, however, that our results are in line with factors found to be important in the literature and hence we would cautiously predict, for example, the factor of sensation-seeking to also manifest in other samples (cf. work in which behavior in an accident situation was predicted by similar personality factors in both a German and a Korean sample^93^).

## Conclusion

In this study we used a VR-based driving simulation to identify personal factors that may be used to predict average and maximum driving speed selection. Our findings indicate that sensation seeking, gender, and age were important factors influencing speed preferences in the sample of drivers tested here. Since driving accidents occur more in rural areas and more than a quarter of fatalities are related to speeding^70^, uncovering factors associated with driving speed is important for analyzing accident prevalence in these environments. Our study showed that even though there was no other traffic, several metrics are able to predict speed selection similarly to other types of driving environments^15–17^, and hence we provide more evidence for the critical influence of personal factors on driving behavior. Overall, our results provide an important baseline that can be linked to speeding behavior in general and may be useful for developing intelligent speed management systems based on human factors which try to efficiently prevent speeding violations on the road. Specifically, our results would make it possible to implement intervention schemes targeted at specific demographics. Depending on local regulations, the government, for example, could mandate the assessment of various psychological metrics for novice drivers and drivers whose license was revoked due to speeding infractions. Following this identification of at-risk demographics, specific interventions could be implemented that aim at increasing awareness of speeding or the use of behavioral interventions, such as driving simulations that contain warning systems to give feedback about speeding^94^. Training programs specifically aimed at novice drivers in other contexts have shown promise of a reduction in crash rates of up to 5%^95^, although care has to be taken that skill-focused training will not result in overconfidence^96,97^. Similar interventions could be introduced for drivers who have to renew their license due to speeding violations—a process, which in Germany, for example, already requires psychological assessment and training when re-acquiring licenses due to severe violations of traffic rules^98^.

Overall, we believe that a driving simulation approach to identifying psychological metrics determining driving behavior and according at-risk populations will be an important additional tool in ensuring road and traffic safety in the future.

## Supplementary Information

Supplementary Information.
