# Supplementary material for: Predicting driving speed from psychological metrics in a virtual reality car driving simulation
Source: Sci Rep. 2022 Jun 16;12:10044. doi: 10.1038/s41598-022-14409-1 (PMC9203461; doi:10.1038/s41598-022-14409-1)
Supplement: Supplementary file 1 — Supplementary Information. [file 41598_2022_14409_MOESM1_ESM.docx]

**Supplementary Results**

Table S1 provides information about the personality differences between groups. Table S2 provides information about correlation between driving speed, maximum driving speed with other psychological metrics from male and female participants as well as split by age. Table S3 provides general information about the participants and their performance in the three training sessions and Table S4 provides multiple linear regression analyses to predict average and maximum driving speed in the training sessions.

First, to compare personality differences between different groups, we used independent two sample t-tests based on gender, age, driving frequency and decision-making in the risk situation. The results show that first, male participants showed significantly lower personal distress (*t*(122) = 5.898, *p*<.001), anxiety (*t*(122) = 2.369, *p*=.019) and significantly higher Machiavellianism (*t*(122) = 2.013, *p*=.046) compared to female participants. Second, there were no personality differences between different age groups. Third, participants with a higher driving experience had a significantly lower personal distress (*t*(122) = 2.996, *p*=.003) and higher sensation seeking  (*t*(122) = 2.301, *p*=.023). Fourth, those who favored a risky decision in the event situation had significantly higher psychopathy (*t*(122) = 2.971, *p*=.004), Machiavellianism (*t*(122) = 2.808, *p*=.006) and lower sensation seeking (*t*(122) = 2.389, *p*=.018). In summary, different groups report different personality metrics - more detailed analyses will be to conducted to investigate how their interplay influences speed selection in particular and driving behavior in general (Table S1).

Second, in the main analysis, different genders showed significantly different average driving speed - similarly, age seemed to affect speed selection as well. To clarify these effects, we split groups and conducted correlational analyses between average driving speed and maximum driving speed with personal factors - this was done since the sample was biased towards male participants, leading to potential issues when running detailed multiple linear regressions. We found that for both male and female participants, sensation seeking significantly correlated with average driving speed (Male: r = 0.26, *p* =.013; Female: r = 0.38, *p* =.037), and only male participants showed a significant correlation between maximum driving speed with sensation seeking (r = 0.42, *p* <.091), Bart score (r = 0.28, *p* =.006) and risky decision-making (r = -0.22, *p* =.091). Additionally, we found that sensation seeking significantly correlated with average driving speed (Age over 25: r = 0.50, *p* =.002; Age under 25: r = 0.26, *p* =.012) and maximum driving speed (Age over 25: r = 0.52, *p* =.001; Age under 25: r = 0.34, *p* <.001) for both age groups. Furthermore, the BART score significantly correlated with average driving speed (r = 0.41, *p* =.014) and maximum driving speed (r = 0.35, *p* =.039) for age over 25, and personal distress (r = -0.24, *p* =.024) significantly correlated with average driving speed for age under 25. Given the danger of TypeII errors, we cautiously conclude that sensation seeking, indeed, is an important overall factor for speed selection and that further experiments will need to follow up on additional factors (Table S2).

Third, since adjustment of VR driving would affect driving speed, we used a repeated measures ANOVA to investigate the potential influence of replicated trials. The result showed that average driving speed significantly changed across trials (F(3) = 7.795, *p* < .001). Based on the results, we ran post hoc tests to compare speeds between the test and three training sessions. The results show that driving speed during the test session was significantly increased compared to the first (*t*(66) = 5.390, *p*<.001) and second training session (*t*(61) = 3.512, *p*<.001) but was not significantly different during the last training session (paired sample t-test: *t*(82) = 0.935, *p* =.353). A second repeated measures ANOVA showed similar results for maximum driving speed (F(3) = 4.878, *p* = .004) with maximum speed during the test session significantly increased compared to all three training sessions: first (paired sample t-test: *t*(66) = 4.225, *p*<.001), second (*t*(61) = 2.924, *p*=.005), third (*t*(82) = 3.559, *p*<.001). In summary, results showed that after the training sessions, participants adjusted to the course and gained confidence to increase driving speed to reach their goal faster (Table S3).

Finally, we used multiple linear regression analyses to determine the factors predicting average and maximum driving speed in the training sessions. Overall, the results shown in Table S4 share findings with the test session prediction results in that age and gender again predicted driving speeds. Given the fact that participants first had to accommodate to the driving simulation, and the resulting increased variability in speed for the first training sessions, the results reported in the main manuscript for the test session should be seen as more robust (Table S4).

|  | Male | Female | Age under 25 | Age over 25 | Does not drive | Drive frequently | Risky decision-making in the event - yes | Risky decision-making in the event - no |
| --- | --- | --- | --- | --- | --- | --- | --- | --- |
| Psychopathy (std) | 35.02 (9.04) | 33.25 (9.30) | 34.78 (9.64) | 34.07 (9.39) | 35.03 (9.57) | 34.12 (8.67) | **31.71 (7.81)** | **36.51 (9.45)** |
| Personal distress (std) | **37.21 (14.36)** | **55.65 (17.07)** | 43.06 (16.73) | 38.67 (17.57) | **46.26 (16.63)** | **37.38 (16.34)** | 40.71 (16.80) | 42.57 (17.23) |
| Sensation seeking (std) | 53.98 (11.41) | 48.95 (15.25) | 52.47 (12.68) | 53.36 (12.56) | **50.16 (13.47)** | **55.28 (11.21)** | **55.95 (11.70)** | **50.54 (12.80)** |
| Machiavellianism (std) | **44.00 (11.81)** | **38.76 (14.52)** | 43.41 (13.27) | 40.86 (11.04) | 42.90 (13.85) | 42.47 (11.53) | **38.90 (11.48)** | **45.25 (12.90)** |
| Anxiety (std) | **31.36 (17.03)** | **39.89 (18.34)** | 32.98 (18.56) | 34.81 (15.42) | 35.56 (17.09) | 31.42 (18.17) | 30.70 (16.88) | 35.38 (18.08) |
| Impulsivity (std) | 34.44 (9.34) | 32.97 (12.94) | 34.07 (10.00) | 34.10 (11.25) | 34.75 (10.98) | 33.41 (9.66) | 33.49 (10.27) | 34.47 (10.41) |

**Table S1.** Personality scales between gender, age over 25 and under, frequent driving or not driving and risky decision-making in the event

|  | | **Male only** | | | | **Female only** | | |
| --- | --- | --- | --- | --- | --- | --- | --- | --- |
|  | | Average driving speed | | Maximum driving speed | | Average driving speed | | Maximum driving speed |
| Driving Distance | 0.01 | | 0.02 | | 0.17 | | 0.13 | |
| Psychopathy | 0.04 | | 0.05 | | 0.24 | | -0.56 | |
| Personal distress | 0.08 | | -0.13 | | 0.15 | | 0.19 | |
| Sensation seeking | 0.26* | | 0.42** | | 0.39* | | 0.23 | |
| Machiavellianism | -0.04 | | 0.07 | | 0.28 | | -0.03 | |
| Anxiety | 0.11 | | 0.01 | | 0.10 | | 0.03 | |
| Impulsivity | 0.35 | | -0.01 | | 0.09 | | -0.06 | |
| Age | -0.05 | | -0.14 | | 0.02 | | -0.26 | |
| BART | 0.16 | | 0.28** | | 0.23 | | -0.06 | |
| Risky  decision-making | -0.18 | | -0.22* | | -0.31 | | -0.16 | |
|  | | **Age over 25** | | | | **Age under 25** | | |
|  | | Average driving speed | | Maximum driving speed | | Average driving speed | | Maximum driving speed |
| Driving Distance | -0.02 | | -0.02 | | 0.09 | | 0.08 | |
| Psychopathy | 0.25 | | 0.12 | | 0.06 | | -0.01 | |
| Personal distress | 0.05 | | -0.10 | | -0.24* | | -0.17 | |
| Sensation seeking | 0.50** | | 0.52** | | 0.26* | | 0.34** | |
| Machiavellianism | 0.14 | | 0.07 | | 0.11 | | 0.05 | |
| Anxiety | -0.03 | | -0.12 | | -0.06 | | 0.01 | |
| Impulsivity | 0.17 | | 0.11 | | 0.02 | | -0.07 | |
| Age | -0.20 | | -0.04 | | 0.16 | | 0.04 | |
| BART | 0.41* | | 0.35* | | 0.14 | | 0.07 | |
| Risky  decision-making | -0.29 | | -0.34* | | -0.16 | | -0.17 | |
| ^*^ p < 0.05, ^**^ p < 0.01.  **Table S2.** Correlations between all personal factors with average driving speed and maximum driving speed for each gender and age over 25 and under 25   \| Success / failure in the first training \| 67/57 \| \| --- \| --- \| \| Success / failure in the second training \| 62/62 \| \| Success / failure in the third training \| 83/41 \| \| Driving speed (first training) – km/h (std) \| 60.68 (8.50) \| \| Driving speed (second training) – km/h (std) \| 61.39 (9.04) \| \| Driving speed (third training) – km/h (std) \| 64.56 (11.14) \| \| Maximum speed (first training) – km/h (std) \| 113.33 (13.75) \| \| Maximum speed (second training) – km/h (std) \| 113.52 (12.42) \| \| Maximum speed (third training) – km/h (std) \| 112.34 (15.24) \|   **Table S3**. Descriptive statistics of three trainings   \|  \| Driving speed - training (first) \| \| \| --- \| --- \| --- \| \|  \| β \| p \| \| Psychopathy \| 0.181 \| .358 \| \| Personal distress \| 0.171 \| .249 \| \| Sensation seeking \| 0.122 \| .369 \| \| Machiavellianism \| 0.081 \| .668 \| \| Anxiety \| 0.007 \| .962 \| \| Impulsivity \| 0.071 \| .646 \| \| Annual distance (km/h) \| 0.033 \| .782 \| \| BART \| 0.332 \| .013^**^ \| \| Age \| 0.031 \| .801 \| \| Risky decision-making \| -0.040 \| .777 \| \| Gender \| -0.178 \| .209 \| \|  \| Driving speed - training (second) \| \| \|  \| β \| p \| \| Psychopathy \| 0.333 \| 0.074 \| \| Personal distress \| -0.183 \| 0.231 \| \| Sensation seeking \| 0.195 \| 0.145 \| \| Machiavellianism \| 0.127 \| 0.492 \| \| Anxiety \| -0.175 \| 0.281 \| \| Impulsivity \| -0.001 \| 0.996 \| \| Annual distance (km/h) \| 0.053 \| 0.673 \| \| BART \| -0.107 \| 0.411 \| \| Age \| 0.076 \| 0.552 \| \| Risky decision-making \| -0,222 \| 0.134 \| \| Gender \| 0.029 \| 0.836 \| \|  \| Driving speed - training (third) \| \| \|  \| β \| p \| \| Psychopathy \| 0.026 \| 0.851 \| \| Personal distress \| -0.212 \| 0.079 \| \| Sensation seeking \| 0.147 \| 0.150 \| \| Machiavellianism \| -0.041 \| 0.777 \| \| Anxiety \| 0.003 \| 0.977 \| \| Impulsivity \| 0.140 \| 0.251 \| \| Annual distance (km/h) \| 0.123 \| 0.228 \| \| BART \| 0.084 \| 0.415 \| \| Age \| -0.193 \| 0.058 \| \| Risky decision-making \| -0.037 \| 0.722 \| \| Gender \| -0.382 \| 0.002^* *^ \| \|  \| Maximum driving speed - training (first) \| \|  \| \|  \| β \| p \| \| Psychopathy \| 0.288 \| 0.158 \| \| Personal distress \| -0.108 \| 0.479 \| \| Sensation seeking \| 0.199 \| 0.155 \| \| Machiavellianism \| -0.114 \| 0.558 \| \| Anxiety \| 0.082 \| 0.578 \| \| Impulsivity \| -0.121 \| 0.447 \| \| Annual distance (km/h) \| -0.051 \| 0.674 \| \| BART \| 0.228 \| 0.094 \| \| Age \| 0.004 \| 0.977 \| \| Risky decision-making \| 0.062 \| 0.671 \| \| Gender \| -0.206 \| 0.159 \| \|  \| Maximum driving speed - training (second) \| \| \|  \| β \| p \| \| Psychopathy \| 0.144 \| 0.443 \| \| Personal distress \| -0.263 \| 0.095 \| \| Sensation seeking \| 0.162 \| 0.235 \| \| Machiavellianism \| 0.094 \| 0.619 \| \| Anxiety \| -0.145 \| 0.380 \| \| Impulsivity \| 0.127 \| 0.401 \| \| Annual distance (km/h) \| 0.023 \| 0.860 \| \| BART \| 0.075 \| 0.574 \| \| Age \| -0.079 \| 0.545 \| \| Risky decision-making \| -0.083 \| 0.582 \| \| Gender \| -0.065 \| 0.649 \| \|  \| Maximum driving speed - training (third) \| \| \|  \| β \| p \| \| Psychopathy \| 0.120 \| 0.391 \| \| Personal distress \| 0.079 \| 0.507 \| \| Sensation seeking \| 0.148 \| 0.147 \| \| Machiavellianism \| -0.028 \| 0.842 \| \| Anxiety \| -0.057 \| 0.623 \| \| Impulsivity \| 0.095 \| 0.430 \| \| Annual distance (km/h) \| -0.05 \| 0.622 \| \| BART \| 0.05 \| 0.624 \| \| Age \| -0.323 \| 0.002 ^* *^ \| \| Risky decision-making \| 0 \| 0.998 \| \| Gender \| -0.411 \| 0.001 ^* *^ \|   ^*^ p < 0.05, ^**^ p < 0.01.  **Table S4** Regression analysis for driving speed during training | | | | | | | | |
